# Investigating the effect of curing temperature on the corrosion resistance of epoxy-based composite coatings for aluminium alloy 7075 in artificial seawater

**DOI:** 10.1039/d3ra04138g

**Published:** 2023-07-11

**Authors:** Arshad Ali Khan, Afzal Khan, Zainab Zafar, Ishaq Ahmad

**Affiliations:** a Department of Mechanical Engineering, University of Engineering and Technology 25120 Peshawar Pakistan engrarshad@uetpeshawar.edu.pk; b National Centre for Physics, Quaid-i-Azam University Campus 45320 Islamabad Pakistan

## Abstract

Araldite LY5052 epoxy resin and Aradur HY5052 hardener were used in a ratio of 100 : 38 to produce composite coatings containing 0.05 proportion of functionalized SiO_2_. Coating samples were cured at curing temperatures of 60, 80, 100, 120, and 140 °C. The results of Fourier Transform Infrared Spectroscopy (FTIR) verified that silica particles were successfully functionalized with methyltrimethoxysilane (MTMS)/3-aminopropyl-triethoxysilane (APTES). The epoxide and Si–O bond peaks in the EHS100 coating were present due to the effective incorporation of functionalized silica (FSiO_2_) particles in the polymeric matrix (epoxy resin). The surface morphology of the bare aluminium alloy AA7075 and EHS100 coating was investigated by Field Emission Scanning Electron Microscopy (FE-SEM). Additionally, corrosion analysis was conducted at room temperature using an electrolytic solution of artificial seawater, prepared according to ASTM standard D1141-98. Charge transfer resistance (*R*_ct_) was shown to increase by 86.43, 92.15, 94.76, 90.65, and 83.96% for EHS60, EHS80, EHS100, EHS120, and EHS140 in comparison to bare AA7075 substrate using electrochemical impedance spectroscopy (EIS) examination. Furthermore, potentiodynamic polarization (PDP) measurements were carried out to determine the corrosion rates, which demonstrated a drop of 55.98, 98.96, 99.37, 98.33, and 50.39% for EHS60, EHS80, EHS100, EHS120, and EHS140, as compared to the bare AA7075 sample. The highest charge transfer resistance (29.77 kΩ) and lowest corrosion rate (0.00078 mm per year) were recorded for EHS100, which reveals that the EHS100 coating has the best anti-corrosion performance and provides the maximum corrosion protection for the aluminium alloy AA7075 substrate.

## Abstract 2

We investigate the effect of curing temperature on the corrosion resistance of epoxy-based composite coatings for aluminum alloy AA7075 in artificial seawater.

## Graphical abstract

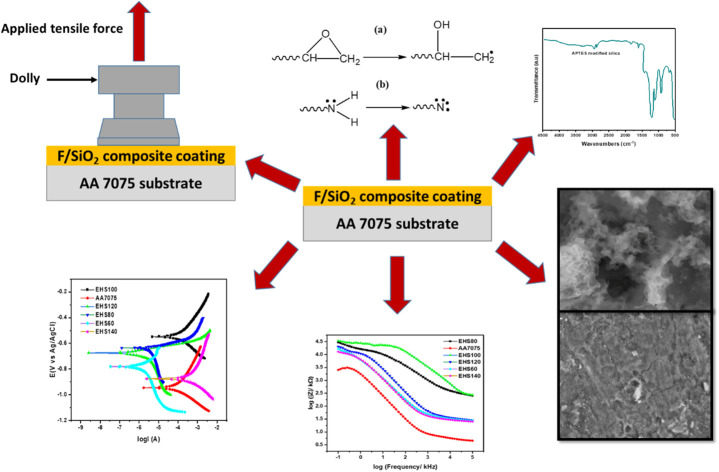

## 1. Introduction

Metallic corrosion especially that of aluminium alloys, is a major issue in a number of industries, including the automotive, marine, and aerospace sectors.^1^ In addition to causing structural integrity to deteriorate, corrosion also causes considerable financial losses. To increase the service life and performance of aluminium alloys in harsh environments, it is crucial to create efficient corrosion prevention measures.^2^ Epoxy-based composite coatings have received a lot of attention among the numerous protective coatings available because of their superior barrier qualities and adhesive strength.^3^ These coatings are frequently employed in industrial settings to offer metallic substrates a protective layer that provides resistance to corrosive, abrasive, and chemical attacks. Araldites are one of the most popular classes of epoxy resins. Four components make up the well-known LY5052/HY5052 epoxy system, which allows for the formation of a variety of epoxy resin systems with a variety of mechanical and thermal properties, such as high-temperature resistance, low viscosity, and simply impregnated reinforcements.^4^ By adding various additives, such as nanoparticles, pigments, and fillers, epoxy-based composite coatings' characteristics can be further improved in order to better suit particular applications.^5,6^ The reinforcement type, orientation, density, interfacial bonding, and surface modification or functionalization have a significant impact on the properties of composite coatings.^7^ Many studies and research have been carried out regarding the functionalization of the fillers used in polymeric resin. Zhang J. *et al.* incorporated functionalized SiN in epoxy resin, to improve the dispersibility of silicon nitride (SiN). The nano-composites were used to coat Q235 carbon steel in order to prevent corrosion. Due to the hydrophobic properties of the modified silicon nitride and its enhanced bonding, the modified silicon nitride coating provided a robust barrier against corrosive electrolytes.^8^ Making high-performance polymer composites requires careful management of the polymer/filler interface interaction. Chen L. *et al.* developed a core–shell structured hybrid (SiO_2_–GO) as a novel filler and added it to an epoxy polymer matrix. Due to the hybrid's integration, the mechanical and anticorrosion characteristics of the epoxy resin were significantly improved by effectively enhancing the interfacial interaction of the epoxy/SiO_2_–GO composites.^9^ The curing temperature used during coating application is one important variable that substantially impacts the functionality of epoxy-based composite coatings. The ultimate composition and characteristics of the coating, which include its mechanical strength, corrosion resistance, and adhesion strength, are profoundly influenced by the curing temperature. The degree of cross-linking between the polymer chains varies with the curing temperature, which further has an impact on the structural integrity of the coating.^10^ Alka Phanasgaonkar *et al.* developed and investigated the silane-based organic–inorganic hybrid coatings with special features that can be applied to steel structures exposed to marine corrosion to enhance their performance. These silane-based sol–gel coatings were synthesized by hydrolyzing and polycondensing methyltriethoxysilane (MTES) and tetraethylorthosilicate (TEOS) under acid catalysis conditions. The coatings were applied on planar samples of mild steel in the solution using the dip coating technique. While curing at 200 °C, coatings were produced without cracks. The plain organic–inorganic hybrid coatings, however, started to break when the curing temperature was raised to 400 °C.^11^ The aluminium alloy (AA2024) substrate surface was coated with modified potassium silicate conversion coatings made of nano-silica. Electrochemical impedance spectroscopy (EIS), potentiodynamic polarisation (PDP), and surface morphology analyzing techniques were used to investigate the corrosion behavior of coatings. It was investigated how curing temperature and duration of curing affected corrosion resistance of coatings. The anti-corrosion properties of silicate conversion coatings were significantly affected by curing temperature, and greater corrosion resistance was attained at 150 °C. Additionally, the experimental results showed that the corrosion resistance substantially increased, when the curing duration for various coatings was elongated.^12^ In the case of aluminium alloy AA7075, which is extensively utilized in marine environments, the corrosion resistance of the substrate can be significantly improved by applying epoxy-based composite coatings.^13^ However, the effect of curing temperature on the corrosion resistance of such coatings for aluminum alloy AA7075 in artificial seawater remains largely unexplored. Understanding this relationship is crucial for optimizing the coating process and enhancing the long-term durability of the coated aluminum alloy.

Therefore, this research paper aims to investigate the effect of curing temperature on the corrosion resistance of epoxy-based composite coatings for aluminum alloy AA7075 in artificial seawater. The corrosion behavior of the coated substrates was evaluated using various electrochemical techniques, such as potentiodynamic polarization (PDP/Tafel) and electrochemical impedance spectroscopy (EIS). The morphology of the samples was investigated using field emission scanning electron microscopy (FE-SEM). Fourier transform infrared spectroscopy was performed for unmodified, MTMS, APTES-modified silica, and EHS100, which confirmed the presence of various functional groups. By systematically altering the curing temperature (60, 80, 100, 120, and 140 °C), this study offers insights into the optimum curing temperature for obtaining excellent corrosion resistance in aluminium alloy AA7075 coated with epoxy-based composites.

## 2. Materials and methods

### 2.1 Chemicals and materials

Epoxy resin, Araldite LY5052, and Aradur hardener HY5052 were purchased from Huntsman. Methyl-ethyl ketone peroxide (MEKPO) and cobalt naphthenate (CN) were used as reaction accelerator and reducing agent respectively. The micro-silica (silica gel 60) of Merck Company was utilized as filler material, having a mesh size of 70–230 and particle size range of 63–200 μm. Silane coupling agents, methyltrimethoxysilane (MTMS), and 3-aminopropyl-triethoxysilane (APTES) were used for the functionalization of silica. The substrate material, wrought aluminium alloy (AA7075) of series 7xxx was provided by Soan Enterprises Islamabad, Pakistan. Artificial seawater according to ASTM standard D1141-98 shown in Table 1, was used as an electrolytic solution.

**Table 1:** “Sea Salt” ASTM D1141-98 composition

Sodium chloride	NaCl	58.490 wt%
Magnesium chloride	MgCl_2_–6H_2_O	26.460 wt%
Sodium sulfate	Na_2_SO_4_	9.750 wt%
Calcium chloride	CaCl_2_	2.765 wt%
Potassium chloride	KCl	1.645 wt%
Sodium bicarbonate	NaHCO_3_	0.477 wt%
Potassium bromide	KBr	0.238 wt%
Boric acid	H_3_BO_3_	0.071 wt%
Strontium chloride	SrCl_2_–6H_2_O	0.095 wt%
Sodium fluoride	NaF	0.007 wt%

### 2.2 Functionalization of silica particles

The dried silica particles were sonicated for 2 hours at 60 °C to disperse in ethanol for functionalization. During the functionalization reaction, pH was kept at 4 to 5 by introducing acetic acid. Adding silane coupling agents (MTMS and APTES) to the silica particle dispersion at a concentration of 5% of the silica's weight, the reaction was allowed to proceed under reflux for 10 hours. The modified solid phase of silica was obtained by centrifugation process and was cleaned with ethanol/DI water for removing residuals if any. In order to remove any remaining water, the particles were then dried for 6 hours at 100 °C.

### 2.3 Preparation of aluminium alloy specimens

The elemental composition of the aluminium alloy AA7075 specimen was (weight%): Si-0.21, Fe-0.24, Cu-1.60, Mn-0.07, Mg-2.42, Cr-0.23, Ni-0.008, Zn-5.59, Ti-0.045, V-0.008 and Al-balance. All specimens (having surface areas of 3 cm^2^) were polished using SiC papers having grit sizes up to 2000. After the polishing step, all the samples were rinsed with DI water and acetone before applying the coatings.

### 2.4 Synthesis of composite coatings

Epoxy resin and hardener with a mixing ratio of 2.632 : 1 (parts by weight, 100 : 38) were used to produce the composite coatings. Functionalized silica and MEKPO having concentrations of 5 × 10^4^ ppm and 1.5% by weight respectively, were added to 5 g of hardener and 13.16 g of epoxy resin in a beaker and stirred mechanically to formulate the composite of epoxy-functionalized micro-silica. A few drops of cobalt naphthenate (reducing agent for MEKPO) were used during the stirring process. Using the dip coating process, the suspension was applied on substrate aluminium alloy AA7075. After this, all the samples were permitted to dry for 4 hours at ambient temperature. Moreover, the samples were then subsequently cured at different curing temperatures; 60, 80, 100, 120 and 140 °C, while allowing curing times of 5, 4, 3, 2, and 1 h respectively. The thickness of all coatings on the substrate aluminium alloy was in the range of 100 ± 10 μm. The bare aluminium alloy AA7075 and composite coatings cured at various temperatures are designated as AA7075, EHS60, EHS80, EHS100, EHS120, and EHS140, and their description is given in Table 2.

**Table 2:** Description of prepared samples

Designated symbols	Details of samples
AA7075	Aluminium alloy substrate
EHS60	Epoxy-hardener ratio 100 : 38, 5 wt% functionalized silica, cured at 60 °C
EHS80	Epoxy-hardener ratio 100 : 38, 5 wt% functionalized silica, cured at 80 °C
EHS100	Epoxy-hardener ratio 100 : 38, 5 wt% functionalized silica, cured at 100 °C
EHS120	Epoxy-hardener ratio 100 : 38, 5 wt% functionalized silica, cured at 120 °C
EHS140	Epoxy-hardener ratio 100 : 38, 5 wt% functionalized silica, cured at 140 °C

### 2.5 Molecular structures of resin and hardener

Epoxy phenol novolac and 1,4-butanediol diglycidyl ether are the two constituents of Araldite LY5052 resin. Aradur HY5052 hardner contains isophorone diamine (IPDA) and cycloaliphatic diamine. A recap of the breakdown is available in Table 3.

**Table 3:** Constituents of epoxy resin Araldite LY5052 and hardener Aradur HY5052

Epoxy resin	Araldite LY5052	(a) Epoxy phenol novolac
		(b) 1,4-Butanediol diglycidyl ether
Amine hardener	Aradur HY5052	(a) Isophorone diamine (IPDA)
		(b) Cycloaliphatic diamine

**Fig. 1:**
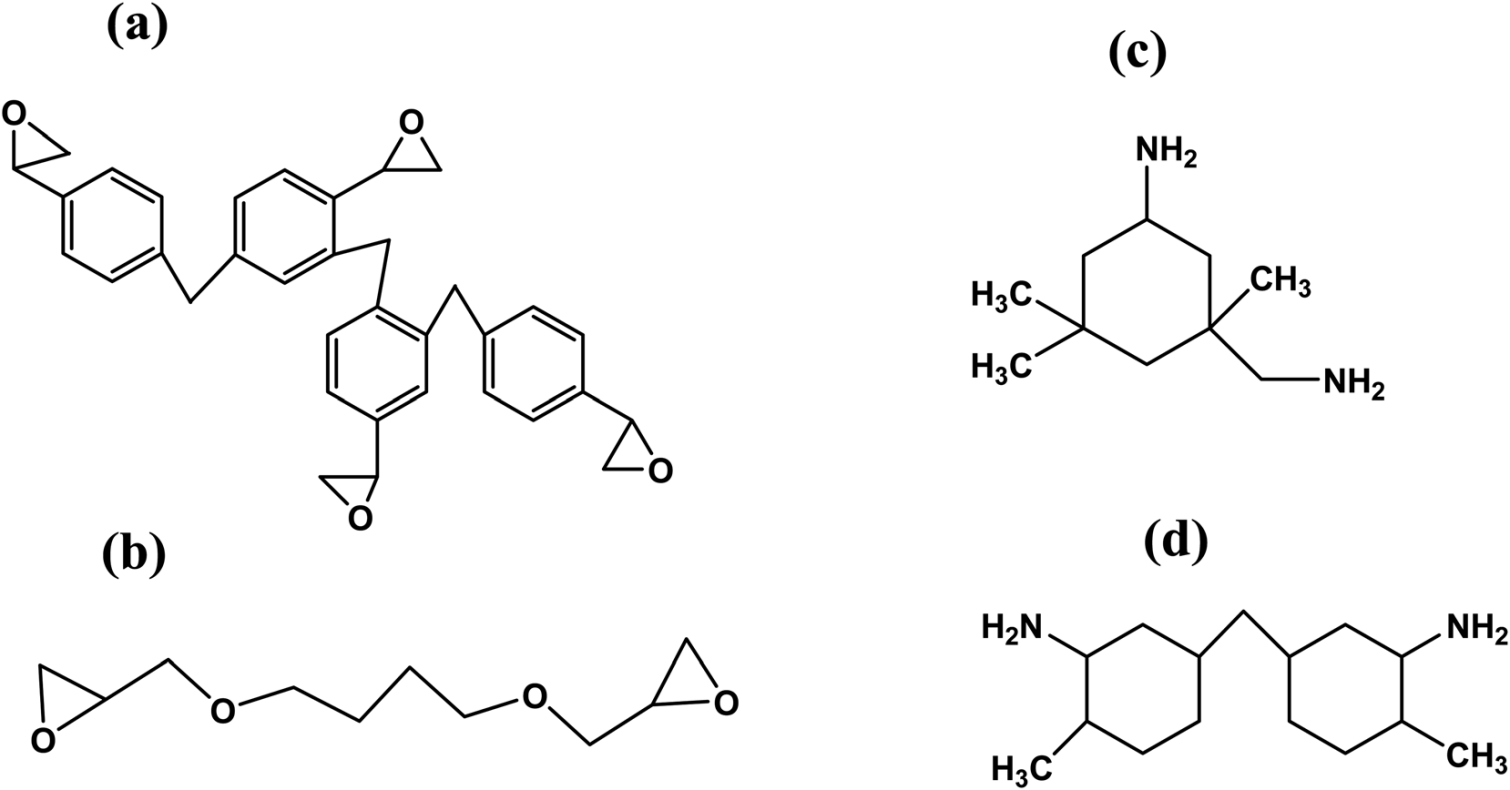
Chemical structures of epoxy resin Araldite LY5052 components: (a) epoxy phenol novolac, (b) 1,4-butanediol diglycidyl ether and Aradur hardener HY5052 components, (c) isophorone diamine, and (d) cycloaliphatic diamine.

Phenol novolac and 1,4-butanediol diglycidyl ether both have two epoxide functional groups, while IPDA and cycloaliphatic diamine both contain two amine functional groups. Epoxide groups in epoxy resin and hardener's amine functional groups bond to produce crosslinked polymer chains. Fig. 1 displays the molecular constitution of each component.

### 2.6 Characterization

#### 2.6.1. Visual inspection

The coating's appearance was checked for color, transparency, homogeneity, and any defects that might have been present.

#### 2.6.2. Fourier transform infrared (FTIR) spectroscopy

The FTIR spectra were obtained using FT/IR-6600 Spectrometer at ambient temperature. The analysis was carried out over a wavelength range of 4000–400 cm^−1^. The spectra were recorded using a resolution of 8 cm^−1^ and a scanning speed of 2 mm s^−1^.

#### 2.6.3. Scanning electron microscopy (SEM)

The surface morphology of the bare aluminium alloy substrate and composite coated samples was investigated by using Field Emission Scanning Electron Microscopy (FE-SEM).

#### 2.6.4. Pull-off adhesion test

The adhesion strength of coatings can easily be measured using adhesion testing, which has gained popularity. The quantitative pull-off test is one of the most widely used techniques for examining adhesion strength. One of the most widely used techniques for quality coating checks is a pull-off test in accordance with ASTM D-4541, which entails adhering the dolly to the solid surface (aluminium alloy AA7075 in this research work) and then pulling the dolly with a force perpendicular to the surface to remove the dolly and the coating from the substrate. Pull-off adhesion tests were conducted to evaluate the coatings' adhesion to the aluminium alloy substrate employing an Automatic F510-20T gauge with a dolly size (*∅* 10 mm), while a tensile force is being applied at a steady rate of 0.8 MPa s^−1^. For each prepared sample, the values of pull-off adhesion strength (MPa) were repeated at least five times, to achieve accurate results.

#### 2.6.5. Electrochemical impedance spectroscopy (EIS)

The electrochemical measurements were carried out using the Gamry potentiostat system as an electrochemical workstation. Bode plots were generated during the EIS tests, keeping the frequency range of 100 000–0.1 Hz and the number of points per decade frequency value as 10 for all samples.

#### 2.6.6. Potentiodynamic (PDP)/Tafel tests

The electrochemical setup, Gamry potentiostat, was used to conduct potentiodynamic tests. Potentiodynamic curves were obtained for all samples in the electric potential range of (−0.1 to 1 V).

## 3. Results and discussion

### 3.1 Methyl ethyl ketone peroxide (MEKPO) decomposition

**Fig. 2:**
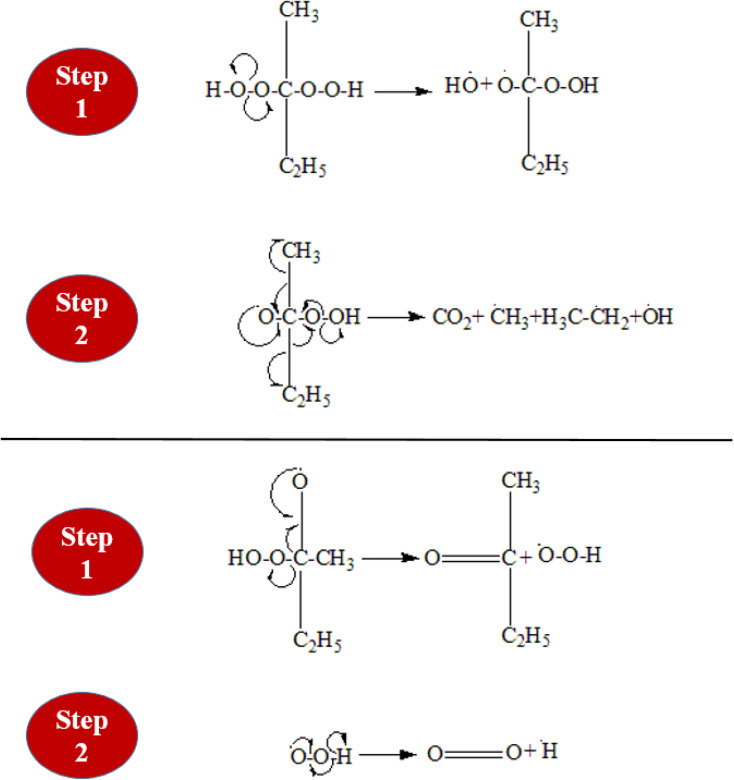
Two decomposition mechanisms of pure MEKPO monomer.

Methyl Ethyl Ketone Peroxide (MEKPO), is a widely used free radical initiator, especially for unsaturated polymeric resins. Free radicals produced by the breakdown of MEKPO start polymerization processes. MEKPO can decompose in a number of ways, but the two main decomposition processes are redox decomposition and thermal breakdown. The decomposition of MEKPO is demonstrated in Fig. 2. When MEKPO is exposed to heat, which may come from an external source or be produced during the curing process, it thermally decomposes. When MEKPO interacts with an appropriate reducing agent, sometimes known as a hardener or activator, it undergoes redox decomposition. Both decomposition mechanisms have three steps; initiation, propagation, and termination. During the initiation of the redox reaction, the reducing agent cobalt naphthenate (cobaltous salt of 2-ethyl hexanoic acid, a major component), reacts with MEKPO, forming a complex.^14^ This complex decomposes quickly and is very unstable. As a result of the complex's homolytic cleavage (decomposition), free radicals such as methyl, ethyl, and peroxy radicals are produced. The produced alkyl and peroxy radicals pull hydrogen atoms from unsaturated monomers to start the polymerization reaction. The reaction propagation continues until all the radicals are consumed with other species (epoxy resin and hardener) and finally, the reaction terminates.

### 3.2 Cross-linking of epoxy-hardener system

**Fig. 3:**
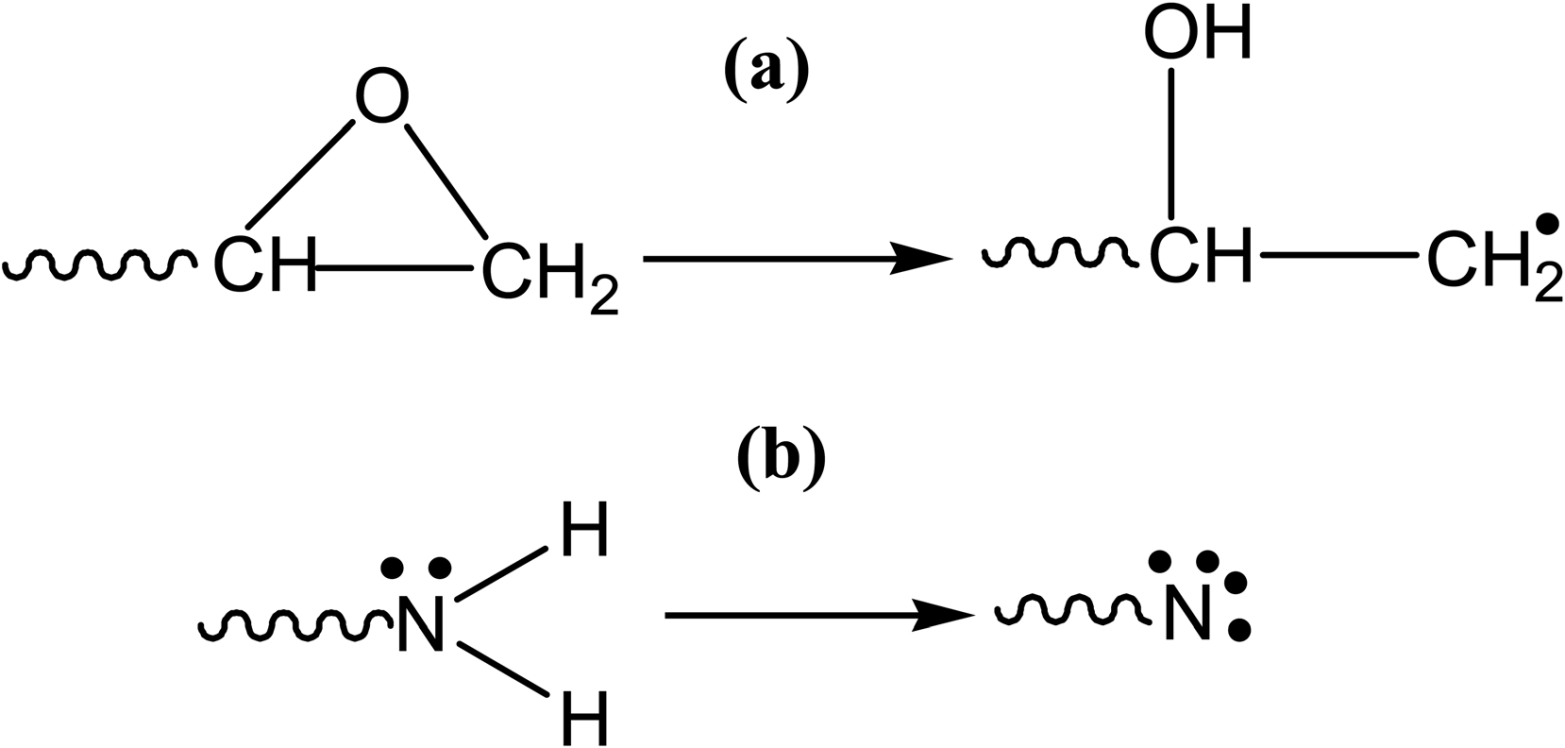
Activation during cross-linking (a) epoxide ring (b) amine group.

Cross-linking is a vital step in the polymerization process. The crosslink density of an epoxy resin system effects its mechanical characteristics, with a higher density resulting in better mechanical properties. The root mean square distance (RMS), called “cut-off distance,” between the carbon atoms in the epoxide ring and N atoms in the amine functional group of the hardener in an equilibrated arrangement provides the basis for cross-linking. Crosslinking causes the simultaneous breakdown of the CH_2_–O and N–H bonds while triggering the CH_2_ and N sites. At an appropriate cutoff distance, activated N and CH_2_ then combine to form a crosslink. The activation of the epoxide ring and amine to crosslink has been shown in Fig. 3. Generally, a crosslink of 100% is impractical. Different lengths of polymer chains could be produced based on the root mean square distance.^15^ Moreover, the incorporated functionalized silica reacts with the epoxy as well during the crosslinking mechanism. A highly cross-linked three-dimensional network is created once the epoxy-hardener liquid mixture containing functionalized silica has been cured.

### 3.3 FTIR spectroscopy analysis

**Fig. 4:**
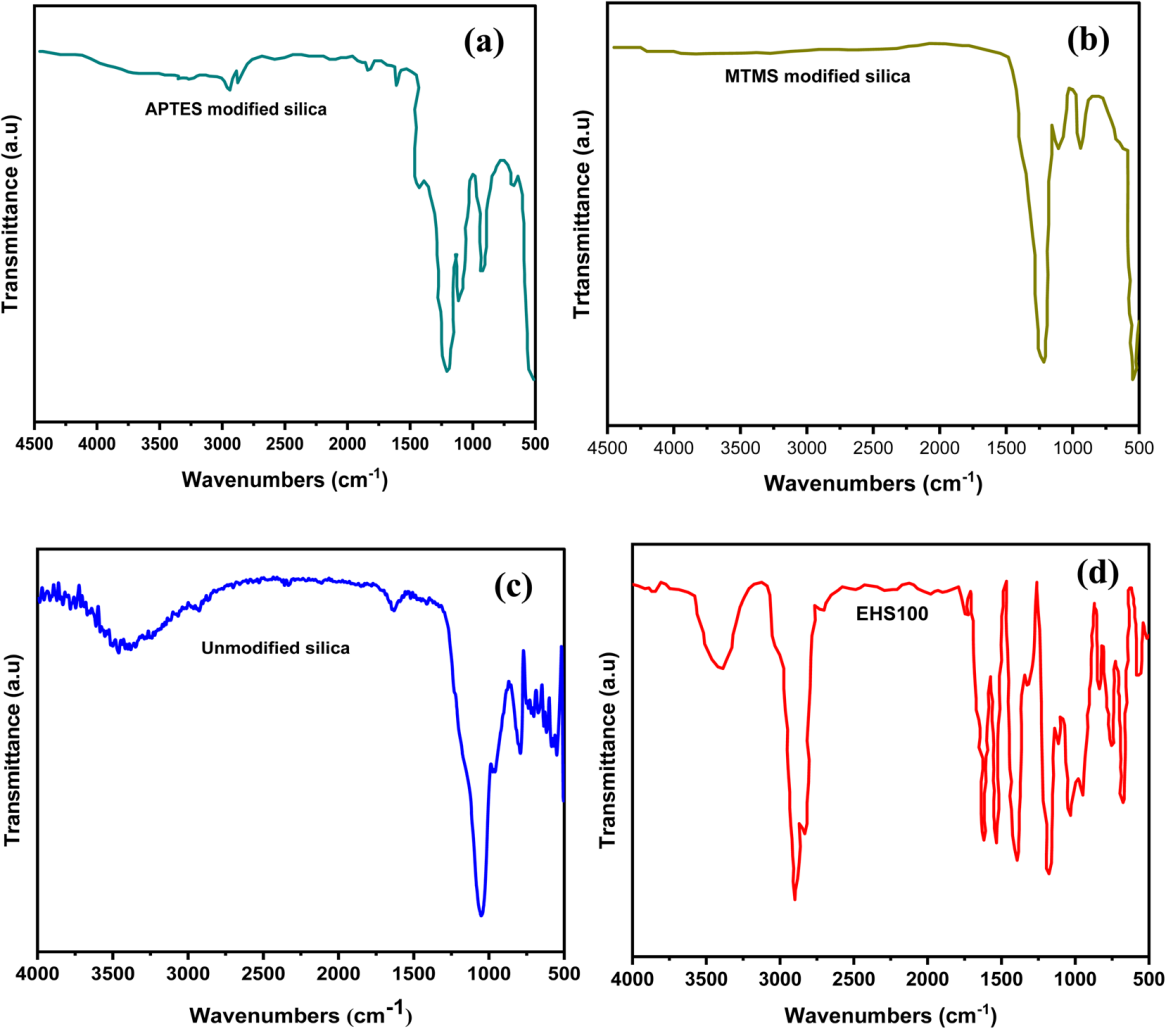
FTIR spectra of (a) APTES-modified silica particles (b) MTMS-modified silica particles (c) unmodified silica particles and (d) EHS100 sample.

By employing FTIR spectroscopy, the unmodified silica,the APTES modified silica and the MTMS modified silica particles and the MTMS modified silica particles were examined. Their spectra were obtained as shown in Fig. 4(c), (a) and (b) respectively. The FTIR spectra of EHS100 were also depicted in Fig. 4(d). The peaks at about 1068 cm^−1^, 935 cm^−1^, and 805 cm^−1^ attributed to asymmetric Si–O, symmetric Si–OH, and symmetric Si–O bonds respectively, verified the silica.^16,17^ No new peaks were seen after modification with MTMS, but after modification with APTES, the peaks positioned at 1615 cm^−1^ and 1427 cm^−1^ appeared and were identified as the NH_2_ deformation modes of the groups of amines. Additionally, peaks observed at 2927 cm^−1^ and 2885 cm^−1^ were linked to the stretching modes of CH_2_, indicating that silica particles had been successfully functionalized with APTES.^18,19^ The Si–O–Si peaks at 790 cm^−1^ and 962 cm^−1^ demonstrate symmetric stretch vibrations and Si–O–Si at 547 cm^−1^ represents the bending vibrations, showing the characteristic of silica.^20^ Moreover, the details of FTIR spectra for EHS100 are shown in Table 4.

**Table 4:** Functional groups present in sample EHS100

Functional groups	Wavenumbers (cm^−1^)
Epoxy group	∼910–1260
Amine group	∼3300–3500
Carbonyl group (C=O stretching)	∼1700–1750
Hydroxyl group (O–H stretching)	∼3200–3600
Silanol group (Si–OH stretching)	∼3600–3700
Siloxane bond (Si–O–Si)	∼1000–1100
Silica network (Si–O–Si)	∼1000–1200

### 3.4 SEM analysis

**Fig. 5:**
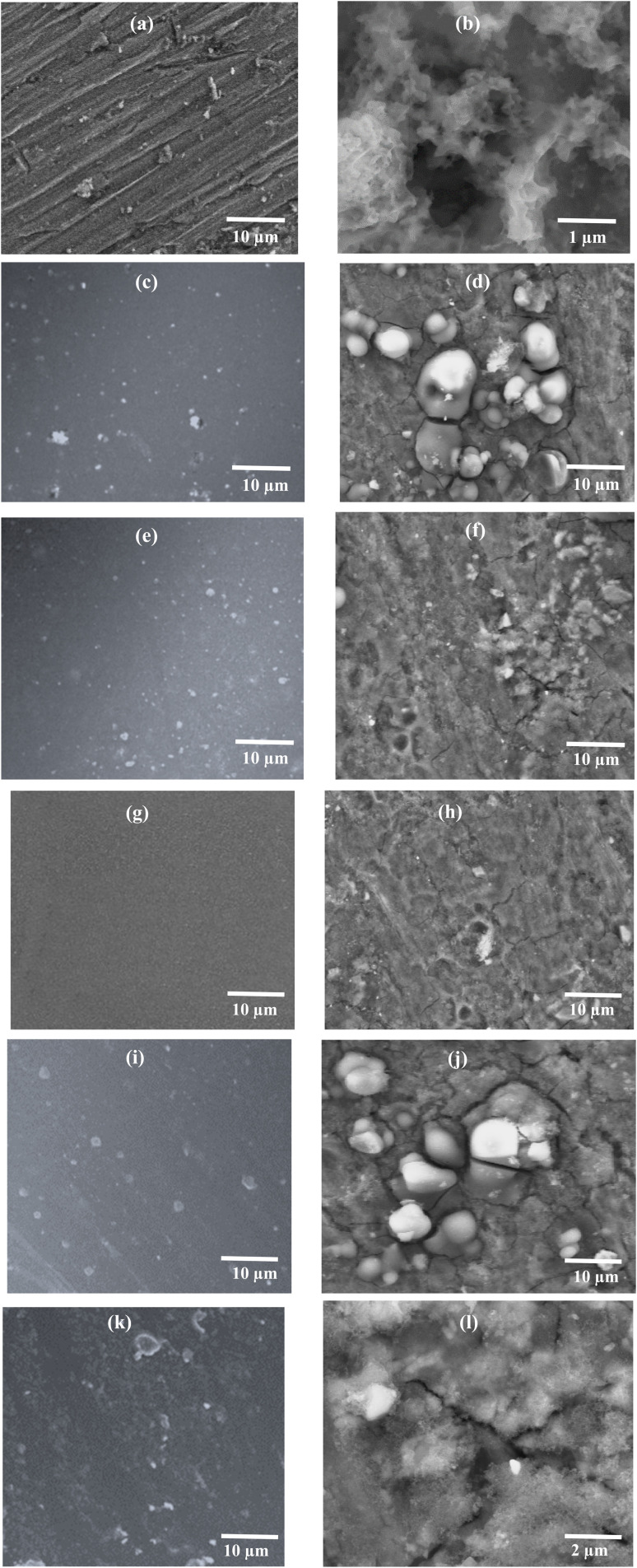
SEM micrographs before the electrochemical tests (a) AA7075 bare substrate (c) EHS60 (e) EHS80 (g) EHS100 (i) EHS120 (k) EHS140, and after the electrochemical tests (b) AA7075 bare substrate (d) EHS60 (f) EHS80 (h) EHS100 (j) EHS120 (l) EHS140.

Fig. 5 shows the SEM micrographs of all the prepared samples (AA7075, EHS60, EHS80, EHS100, EHS120, and EHS140), before and after electrochemical tests for comparison. Fig. 5(a) and (b) show the surface of AA7075 bare substrate before the electrochemical test and AA7075 bare substrate after the electrochemical test. It is clear from Fig. 5(b) that the surface of the bare aluminium alloy 7075 specimen has degraded the most and corrosion products are formed on it. The direct contact of the alloy surface with the electrolytic solution promotes the corrosion phenomenon. Fig. 5(g) and (h) show the surface morphology of EHS100 (composite coating containing 5 wt% of functionalized SiO_2_ particles and cured at 100 °C). It is obvious that the surface is homogeneous and has no irregularities even after electrochemical tests, which reveals the perfect application of the coating on the substrate surface. Further, the presence of bright spots shows that functionalized SiO_2_ particles are uniformly dispersed in the coating. Due to this, EHS100 coating exhibits the highest corrosion protection efficiency, while playing a great role in the corrosion inhibition process. Based on the obtained micrographs, the order of corrosion protection performance for the samples is EHS100 > EHS80 > EHS120 > EHS60 > EHS140 > AA7075.

### 3.5 Pull-off adhesion strength analysis

**Fig. 6:**
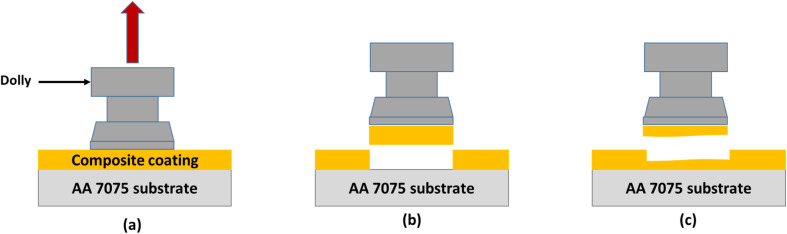
Pull-off adhesion test (a) application of tensile force on the dolly (b) adhesive failure at interface of substrate/coating (c) cohesive failure within layers of coating.

**Fig. 7:**
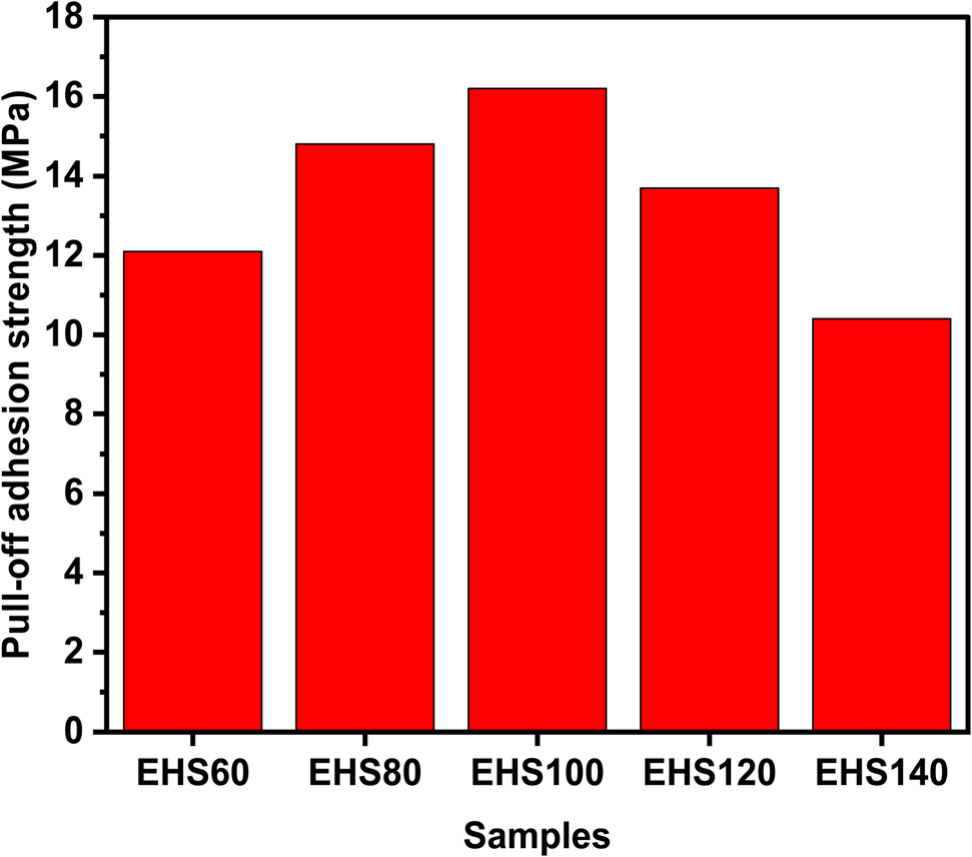
Comparison among the pull-off adhesion strengths for prepared samples.

There are two types of failures that occur during the pull-off adhesion test. Failure can either be a result of a failure occurring between the layers of the particulate composite or it can be due to the failure at the interface of the substrate aluminium alloy/coating as depicted in Fig. 6. The failure between coating layers is due to the adhesive force of the composite/substrate being greater than the cohesive force of the coating, while adhesive failure is due to the inverse relationship. The pull-off adhesion strength is an essential indicator in determining how well a coating adheres to its substrate. Several variables are at play when comparing the pull-off adhesion strength of silica-based epoxy coatings cured at various temperatures (60 °C, 80 °C, 100 °C, 120 °C, and 140 °C). In general, raising the epoxy coating's curing temperature can improve the pull-off adhesion strength. Greater cross-linking and curing of the epoxy resin are made possible by higher temperatures, which enhance the interfacial connection between the epoxy resin and the substrate.^21^ This trend is observed for EHS60, EHS80, and EHS100 as demonstrated in Fig. 7. EHS100 has the highest pull-off adhesion strength (16.2 MPa). There is a non-linear correlation between curing temperature and adhesion strength. For the samples EHS120 and EHS140, a decrease in pull-off adhesion strength is observed. The lowest value of pull-off adhesion strength (10.4 MPa) is recorded for EHS140. Similarly, for each distinct epoxy composition, there is an optimum curing temperature range where the adhesive strength is maximized. The deviation from this range may result in adverse consequences like decreased adhesion due to inadequate curing or enhanced brittleness.^22^

### 3.6 Electrochemical anticorrosion performance of composite coatings

**Fig. 8:**
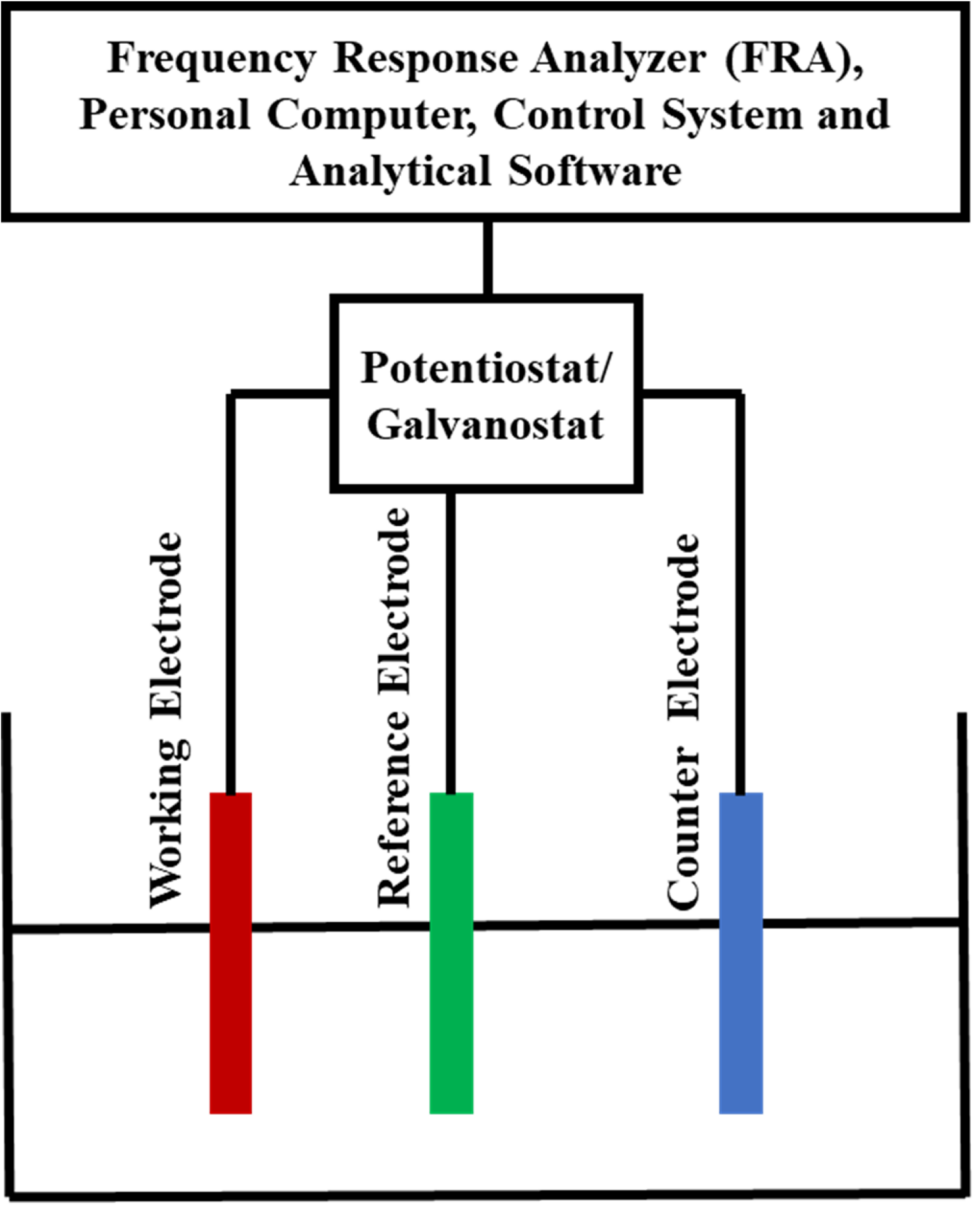
Three electrodes electrochemical cell for corrosion assessment.

For checking the electrochemical anticorrosion performance of the prepared samples, three electrodes electrochemical arrangement has been used as shown in Fig. 8. The electrochemical cell has three electrodes, consisting of saturated Ag/AgCl as the reference electrode (RE), platinum as the counter electrode (CE), and prepared sample as the working electrode (WE). The tests were repeated at least five times for each measurement in order to produce the results and evaluate the performance of the experimental setup.

#### 3.6.1. EIS measurements

**Fig. 9:**
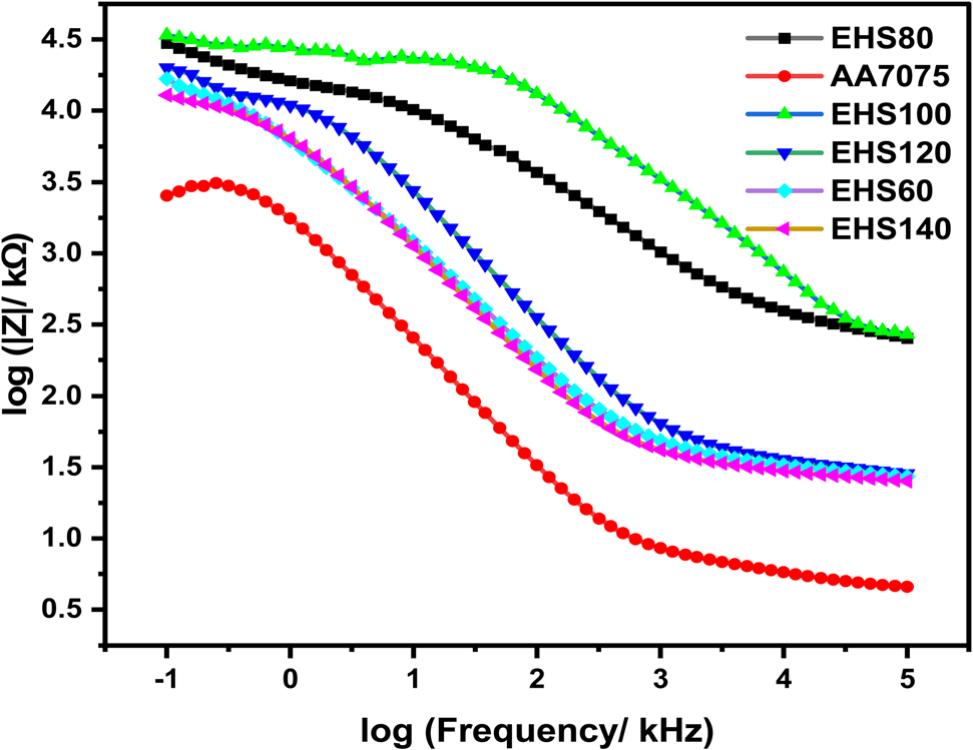
Bode curves obtained from electrochemical impedance spectroscopy for specimens cured at various temperatures measured in artificial seawater.

The electrochemical impedance spectroscopy (EIS) technique was applied to assess the electrochemical behavior of aluminium alloy AA7075 and composite coatings cured at different temperatures in artificial seawater. The Bode plots (log impedance modulus *vs.* log frequency) are shown in Fig. 9. It is obvious in Fig. 9 that the impedance modulus for EHS100 at a lower frequency, *i.e.* |*Z*|_0.01 Hz_ has the highest value during immersion than the remaining coatings.

**Fig. 10:**
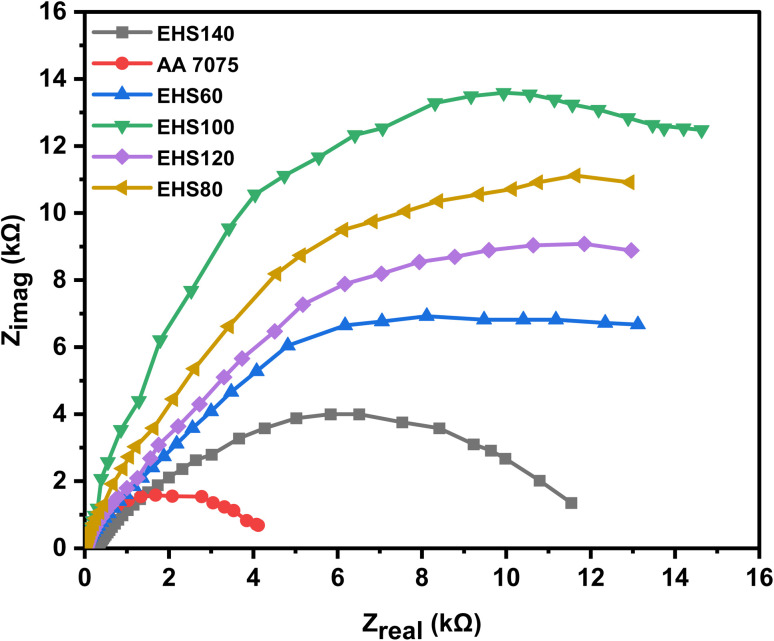
Nyquist plot obtained from electrochemical impedance spectroscopy for samples cured at different temperatures measured in artificial seawater.

The Nyquist plots (real impedance *vs.* imaginary impedance) are displayed in Fig. 10. When considering the corrosion resistance increase of silica-based epoxy coatings cured at different temperatures (60 °C, 80 °C, 100 °C, 120 °C, and 140 °C), Nyquist plots provide insights into the coating's protective performance. As the curing temperature of the silica-based epoxy coating increases, the resulting Nyquist plots exhibit changes in the semicircular arc.^23^ An increase in curing temperature (60 °C, 80 °C, 100 °C) leads to improved coating quality and enhanced corrosion resistance possibly due to better cross-linking and improved adhesion to the substrate alloy. EHS100 demonstrated the best anti-corrosion performance due to these improved properties. The functionalized silica, epoxy resin, and curing agents react more efficiently at the curing temperature of 100 °C. As a result, the coating's ionic or electronic conductivity is reduced, and the charge transfer resistance (*R*_ct_) is increased, which finally leads to the improvement in the coating's corrosion resistance. Due to further increases in curing temperature (120 °C, and 140 °C), a shift toward smaller semicircular arcs and decreased charge transfer resistance in the Nyquist plots indicate the reduction in the corrosion resistance of the silica-based epoxy coatings.

**Fig. 11:**
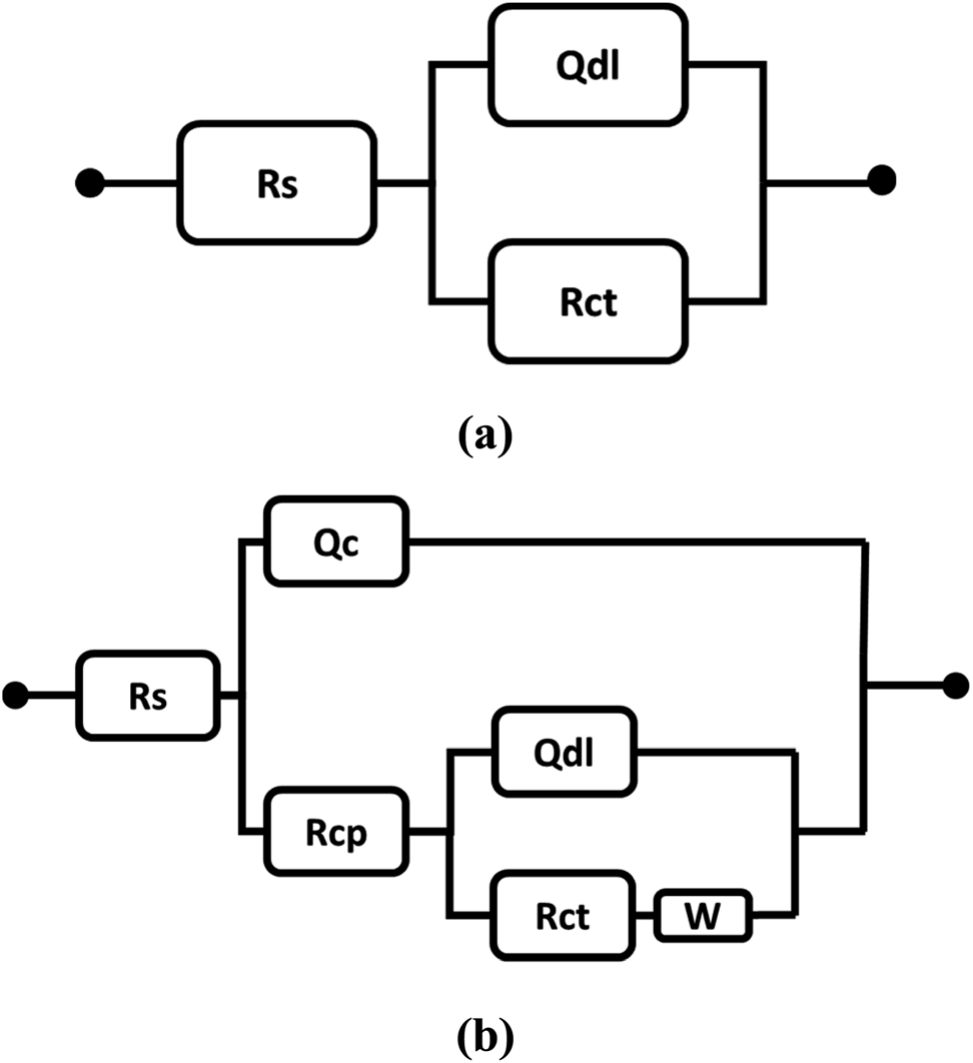
The proposed electrochemical equivalent circuits (a) aluminium alloy AA7075 and (b) composite coatings.

The electrochemical equivalent circuits (EECs) presented in Fig. 11, were used for fitting the EIS data in the Echem Analyst software environment. The EECs comprise solution resistance (*R*_s_), double layer constant phase element (*Q*_dl_), charge transfer resistance (*R*_ct_), coating constant phase element (*Q*_c_), coating pore resistance (*R*_cp_), and Warburg impedance element (*W*). The Warburg impedance element is mentioned in the EECs when the diffusion effect governs the corrosion at a lower frequency for composite coatings.^24^ The inclusion of the Warburg element in the EEC symbolizes the diffusion-controlled reactions at the alloy/coating interface.^25^ It seems that EHS100 coating acts as a protective barrier, which encumbers the diffusion of corrosive ions at the alloy/coating interface.^26^

*R*_cp_ describes the measure of coating degradation and porosity, while *R*_ct_ shows the electric resistance at the alloy/coating interface against the transfer of electrons.^27^ The values of *R*_ct_, *R*_cp_, and other parameters are mentioned in Table 5.

**Table 5:** Bode's plot parameters for substrate AA7075 and composite coatings in artificial sea water

Samples	EIS parameters				
	*Q*_dl_ (μF)	*R*_ct_ (kΩ)	*R*_s_ (Ω)	*Q*_c_ (μF)	*R*_cp_ (Ω)
AA7075	19.54 ± 0.414	1.5610 ± 0.030	5.2578 ± 0.071	—	—
EHS60	15.43 ± 0.331	11.503 ± 0.042	31.89 ± 0.871	9.057 ± 0.062	1152 ± 1.536
EHS80	8.17 ± 0.264	19.880 ± 0.621	53.30 ± 0.921	1.868 ± 0.012	2682 ± 1.812
**EHS100**	**0.9871 ± 0.031**	**29.770 ± 0.920**	**253.3 ± 0.991**	**0.1581 ± 0.011**	**2965 ± 1.521**
EHS120	13.20 ± 0.361	16.704 ± 0.212	32.81 ± 0.910	8.519 ± 0.051	1601 ± 1.201
EHS140	16.39 ± 0.382	9.731 ± 0.132	29.62 ± 0.785	11.026 ± 0.093	568.5 ± 1.132

When functionalized silica (5 wt%) is added to the epoxy coatings and perform coating curing at elevated temperatures, *R*_cp_ and *R*_ct_ values noticeably increase while their *Q*_dl_ and *Q*_c_ values drop with respect to bare aluminium alloy AA7075, as shown in Table 3. It is very clear that composite coatings have a substantially stronger ability to prevent corrosion at elevated temperatures. The increase in *R*_cp_ and *R*_ct_ is more significant when 5 wt% functionalized silica is incorporated in epoxy coating and cured at 100 °C. The substantial improvement in *R*_cp_ against ionic transfer through the EHS100 coating can be attributed to the enhanced cross-link density of epoxy caused by the formation of Si–C bonds within the epoxy-hardener. In addition, significantly higher *R*_ct_ values for EHS100 coatings demonstrate that the penetration of corrosive ions through the coating/metal interface has been restricted. The significant barrier effect is due to the functionalized silica in the coating matrix, and Si–O chemical bonds at the alloy/coating interface are formed, which contribute to improved coating adhesion with the alloy substrate.

Generally, the impedance modulus |*Z*|, at low frequency (|*Z*|_0.01 Hz_) is used to reckon the corrosion resistance of the materials/coatings, while higher values of |*Z*|_0.01 Hz_ advises greater corrosion protection performance.^28,29^

#### 3.6.2. Potentiodynamic measurements

**Fig. 12:**
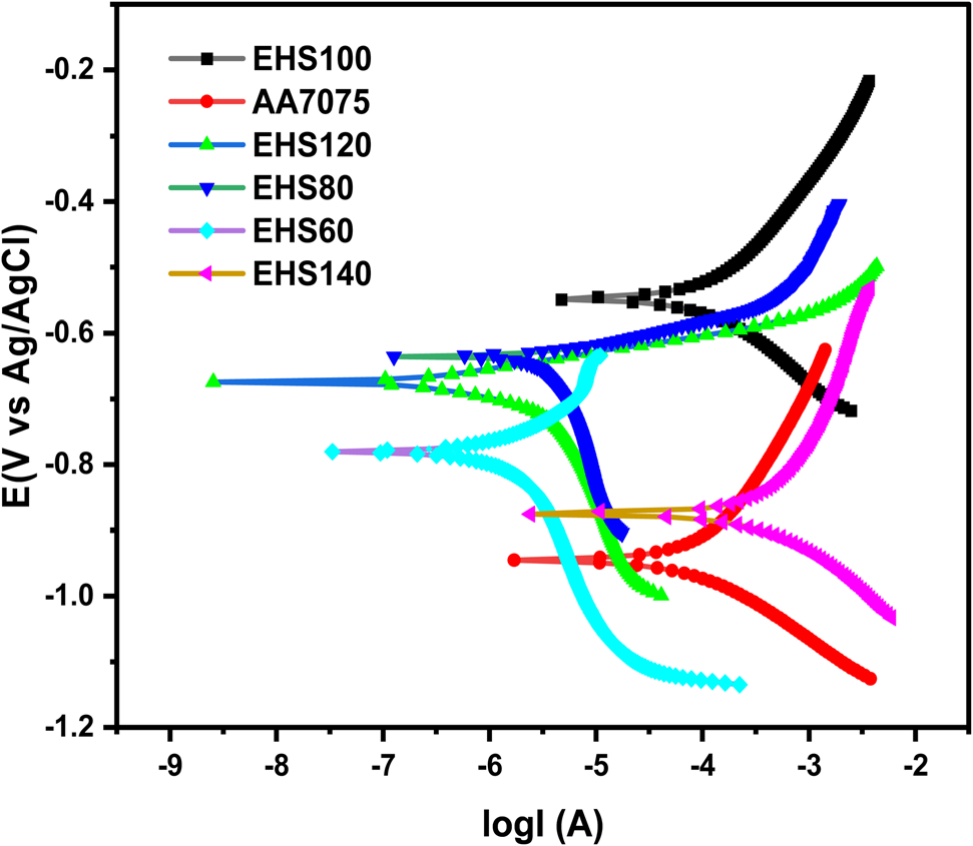
Tafel's curves obtained for specimens cured at various temperatures measured in artificial seawater.

The open circuit potential (*E*_ocp_) is termed the corrosion potential (*E*_corr_), till the potential gains an equilibrium for around 20 minutes. The log *I vs.* log *E* Tafel plots were obtained for a potential range of (−0.1 to 1 V) with respect to *E*_ocp_, as depicted in Fig. 12. The composite coated samples demonstrate a more positive corrosion potential as compared to the bare aluminium alloy specimen. As the curing temperature rises from 60 to 100 °C, the hyperbolic curves move towards lower current densities, which exhibits the anti-corrosion performance of composite coatings cured at 60, 80, and 100 °C. When the curing temperature of the coatings further increased, *i.e.* 120 and 140 °C, *E*_corr_ values become more negative. The hyperbolic curves move towards lower current density values as a result of further temperature increase beyond 100 °C, which demonstrates that the curing temperature has a significant effect on the anti-corrosion behavior of composite coatings. By drawing a straight line through the linear component of the cathodic or anodic plot and extrapolating it to the *E*_corr_ axis, the corrosion current (*I*_corr_) is calculated from the Tafel plot. By applying the Stern–Geary eqn (1), *R*_p_ values are achieved.^30,31^1
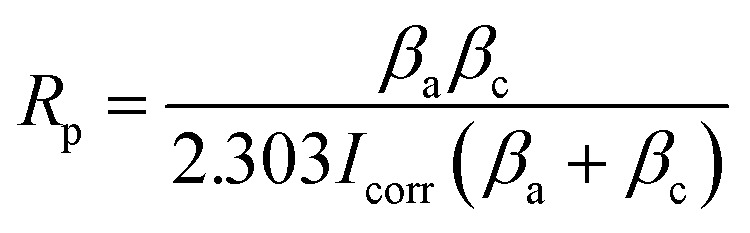
where *β*_a_ and *β*_c_ are anodic and cathodic Tafel constants respectively, *I*_corr_ is the corrosion current (μA). Moreover, using eqn (2), the values of corrosion rates in mm per year are determined.^32–34^2
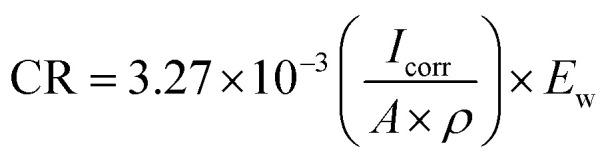
where *E*_w_ is the equivalent weight (g), *ρ* is the density (g cm^−3^) and *A* is the surface area (cm^2^) of the sample. The corrosion protection efficiency P.E.%, indicates the anti-corrosion performance of the coated samples, and its values are calculated using eqn (3).^35^3
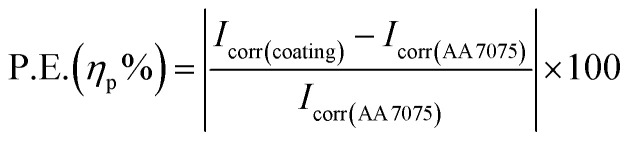
where *I*_corr(AA7075)_ and *I*_corr(coating)_ are the corrosion currents of the aluminium alloy AA7075 and coating respectively.

**Fig. 13:**
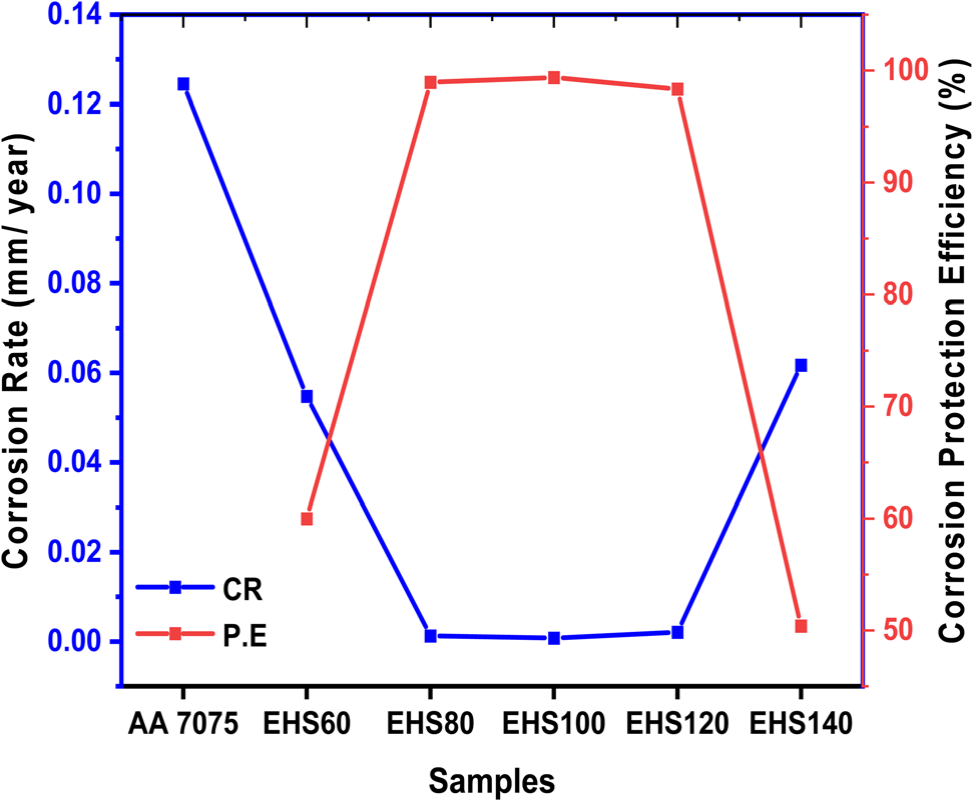
Trend of corrosion rate and corrosion protection efficiency for prepared samples.

Table 6 contains a complete list of all the experimental findings, which were derived from the Tafel plots. Specimen AA7075 has more negative *E*_corr_ (−946 mV) and the highest *I*_corr_ value (33.5 μA), while sample EHS100 has more positive *E*_corr_ (−548 mV) and lowest *I*_corr_ value (0.21 μA). The highest corrosion protection efficiency (P.E.%) value (99.37%) was recorded for EHS100. Moreover, the values of corrosion rates were 0.12450 mm per year and 0.00078 mm per year for AA7075 and EHS100 respectively. The trend of the corrosion rate and corrosion protection efficiency for all the samples has been shown in Fig. 13. These results show that the bare aluminium alloy was protected from corrosion by using composite coatings cured at 100 °C. The change in the values of *E*_corr_, *I*_corr_, CR, and P.E. (%) provides confirmation that EHS100 outpaces the anti-corrosion performance of coatings cured at other temperatures.

**Table 6:** Tafel's plot parameters for substrate AA7075 and composite coatings in artificial sea water

Samples	−*E*_corr_ (mV)	*I*_corr_ (μA)	*β*_a_ (mV per decade)	−*β*_c_ (mV per decade)	CR (mm per year)	P.E.%
AA7075	946 ± 0.55	33.50 ± 0.45	290	122.9	0.12450 ± 0.00215	—
EHS60	781 ± 0.60	14.75 ± 0.20	235.6	114.6	0.05481 ± 0.00320	60.00 ± 1.5
EHS80	636 ± 0.52	0.35 ± 0.01	211.7	98.4	0.00130 ± 0.000981	98.95 ± 0.02
**EHS100**	**548 ± 0.50**	**0.21 ± 0.01**	**65.4**	**35.3**	**0.00078 ± 0.000076**	**99.37 ± 0.01**
EHS120	674 ± 0.54	0.56 ± 0.015	221.1	104.5	0.00208 ± 0.00052	98.33 ± 0.02
EHS140	875 ± 0.56	16.62 ± 0.35	268.5	119.2	0.06176 ± 0.00212	50.39 ± 1.5

## 4. Conclusions

An efficient approach of integrating organosilanes and inorganic fillers was presented to improve the silica dispersion in the polymeric coating system of Araldite epoxy resin LY5052 and Aradur hardener HY5052 cured at different temperatures, which was employed for corrosion protection of aluminium alloy AA7075 substrate.

The conclusions listed below can be presented systematically based on the obtained results:

(1) When compared to the bare aluminium alloy AA7075, the charge transfer resistances (*R*_ct_) of EHS60, EHS80, EHS100, EHS120, and EHS140 were 7.37, 12.74, 19.07, 10.70, and 6.23 times improved. Similarly comparing EHS60, EHS80, EHS100, EHS120, and EHS140 to bare AA7075, the corrosion rates (CR) were decreased by 2.27, 95.77, 159.62, 59.86, and 2.02 folds, respectively. The highest charge transfer resistance (29.77 kΩ) and lowest corrosion rate (0.00078 mm per year) were recorded for EHS100, which exhibits that EHS100 coating has the best anti-corrosion performance and provides the maximum corrosion protection for the aluminium alloy AA7075 substrate.

(2) The resistance to corrosion of composite (epoxy/FSiO_2_) coatings increased with increasing the curing temperature demonstrated by EIS and potentiodynamic polarization tests, due to the creation of strong bonds of Si–O–C and Si–O–Si, which possibly leads to significant improvement in the cross-linked density of the three dimensional cured structure of the final dry composite coating.

(3) The modified functionalized silica dispersed in the samples cured at various temperatures considerably effected the barrier performance and contributed to the surface corrosion resistance of the coatings by making the diffusion reaction of the electrolytic solution (artificial seawater) harder.

The findings of this research work not only advance knowledge of the relationship between curing temperature and corrosion resistance of functionalized silica-based polymeric coatings but also provide beneficial recommendations for the synthesis and use of epoxy/FSiO_2_ coatings for aluminium alloys in harsh marine applications. A more durable and prolonged service life for aluminium alloy components in numerous industries may result from this research's contribution and also leads to the development of a more effective corrosion protection technique.

## Author contributions

Arshad Ali Khan: conceptualization, methodology, validation, formal analysis, investigation, writing – original draft, visualization, writing – review & editing; Afzal Khan: supervision; Zainab Zafar: co-supervision; Ishaq Ahmad: project administration.

## Conflicts of interest

There are no conflicts of interest to declare.

## Supplementary Material
